# Application of Certain *π*-Acceptors for the Spectrophotometric Determination of Alendronate Sodium in Pharmaceutical Bulk and Dosage Forms

**DOI:** 10.1155/2011/680902

**Published:** 2011-06-28

**Authors:** Asad Raza, Muhammad Zia-ul-Haq

**Affiliations:** ^1^Analytical Laboratory, Bio Fine Pharmaceuticals, 74 Industrial Estate, Multan 60900, Pakistan; ^2^Department of Pharmacognosy, Research Institute of Pharmaceutical Sciences, University of Karachi, Karachi 75270, Pakistan

## Abstract

Two simple, fast, and accurate spectrophotometric methods for the determination of alendronate sodium are described. The methods are based on charge-transfer complex formation of the drug with two *π*-electron acceptors 7,7,7,8-tetracyanoquinodimethane (TCNQ) and 2,3-dichloro-5,6-dicyano-1,4-benzoquinone (DDQ) in acetonitrile and methanol medium. The methods are followed spectrophotometrically by measuring the maximum absorbance at 840 nm and 465 nm, respectively. Under the optimized experimental conditions, the calibration curves showed a linear relationship over the concentration ranges of 2–10 *μ*g mL^−1^ and 2–12 *μ*g mL^−1^, respectively. The optimal reactions conditions values such as the reagent concentration, heating time, and stability of reaction product were determined. No significant difference was obtained between the results of newly proposed methods and the B.P. Titrimetric procedures. The charge transfer approach using TCNQ and DDQ procedures described in this paper is simple, fast, accurate, precise, and extraction-free.

## 1. Introduction

Alendronate sodium is sodium trihydrogen (4-amino-1-hydroxybutylidene) biphosphonate trihydrate. It belongs to the bisphosphonate group and is used for the treatment of Paget's disease of bone and osteoporosis, it diminishes bone resporption and thus reduces bone turnover [1, 2]. 

A survey of literature revealed that there are very few methods available for the determination of alendronate sodium. These methods include spectrophotometric [3–6], chromatographic [7–13], capillary electrophoresis [14], inductively coupled plasma [15], and voltammetric [16]. These previously reported spectrophotometric methods in the literature suffer from disadvantages like extraction, long time for the reaction to complete, narrow range of determination, and lack of sensitivity.

Spectrophotometric techniques continue to be the most preferred methods for routine analytical work due to their simplicity and reasonable sensitivity with significant economical advantages. On the other hand, it is well known that *p*-benzoquinones such as 7,7,7,8-tetracyanoquinodimethane (TCNQ) and 2,3-dichloro-5,6-dicyano-1,4-benzoquinone (DDQ) as *π*-electron acceptors often form highly colored electron-donor-acceptor (EDA) or charge transfer (CT) complexes with various donors which provides the possibility of determination of drugs by spectrophotometric methods [17, 18]. In order to develop new simple, fast, and extraction-free spectrophotometric methods for the determination of alendronate sodium in pure and pharmaceutical dosage forms, we investigated quantitative reactions of alendronate sodium with organic *π* -acceptors like TCNQ and DDQ.

The aim of the present paper is to develop the simple and accurate spectrophotometric methods for the determination of alendronate sodium that permits its analysis in dosage forms without interference from excipients and other coformulated drugs.

## 2. Experimental

### 2.1. Apparatus

All spectrophotometric measurements were carried out using a spectrophotometer (U 1100 Hitachi, Japan) with silica glass cell of 1 cm thickness. Officially, calibrated Pyrex glassware was used throughout this study.

### 2.2. Materials

Alendronate sodium was supplied by Indus Pharma (Pvt.) Ltd., Karachi, Pakistan. Standard stock solution of alendronate sodium was prepared by dissolving 50 mg pure drug in 20 mL methanol and volume was diluted to the mark in 100 mL calibrated flask with acetonitrile, for method A and with methanol for method B.

### 2.3. Reagents

All reagents and solvents used were of Analytical Reagent Grade. 7,7,8,8-tetracyanoquinodimethane (TCNQ) (*Fluka, *Austria) 1.0 mg mL^−1^ solution was prepared in acetonitrile, and 2,3-dichloro-5,6-dicyano-1,4-benzoquinone (DDQ) (*Fluka, *Switzerland) 5.0 mg mL^−1^ solution was prepared in methanol.

### 2.4. Assay Procedure

#### 2.4.1. Recommended Assay Procedure

**Method A**

Aliquots of alendronate sodium (2–10 *μ*g mL^−1^) were pipetted into a series of 10 mL standard measuring flasks. A one  mL volume of 1 mg mL^−1^ solution of 7,7,8,8-tetracyanoquinodimethane (TCNQ) was added to each flask and the contents were heated at 70°C in water bath for five minutes. Cooled and diluted the content up to the volume with acetonitrile. The colored product, formed and remained stable for 24 hours. The absorbance was measured within the stability period after dilution at 840 nm against reagent blank prepared simultaneously.

**Method B**

Aliquots of alendronate sodium (2–12 *μ*g mL^−1^) were pipetted into a series of 10 mL standard measuring flasks. A 1.2 mL volume of 5 mg mL^−1^ solution of 2,3-dichloro-5,6-dicyano-1,4-benzoquinone (DDQ) was added to each flask and the contents were diluted to the volume with methanol. The colored product formed immediately and remained stable for two hours. The absorbance was measured within the stability period after dilution at 465 nm against reagent blank.

#### 2.4.2. Assay Procedure for Tablets

Twenty tablets were accurately weighed and powdered. A portion equivalent to 50 mg of alendronate sodium was stirred with 20 mL methanol. The sample solution was filtered using Whatman filter paper number 42. The residue was washed with methanol or acetonitrile according to the method. The filtrate and washings were diluted with appropriate solvent to 100 mL using a measuring flask. The aliquot from this filtered solution was analyzed using the recommended procedures.

### 2.5. Determination of the Molar Ratio

The Job's method of continuous variation was employed. Master equimolar solutions of the drug and color producing reagent were prepared. The concentration of the drug solution was 5 *μ*g/mL. A series of 10 mL portions of the master solutions of the drug with color producing reagent reagent were made up comprising different complementary proportions (0 : 10, 1 : 9,…, 9 : 1, 10 : 1) in 10 mL volumetric flasks. The reactions were allowed to proceed at specified temperatures depending upon the methods. The absorbances of the solutions were measured at wavelengths 840 nm and 465 nm against the reagent blank.

## 3. Results and Discussion

### 3.1. Involved Reaction and Absorption Spectra

The reaction involved in the present study was based on charge transfer basis. TCNQ is used for quantitative determination of pharmaceutical drugs in dosage forms by charge-transfer complex formation [19, 20]. Interaction of alendronate sodium with TCNQ in acetonitrile solution was found to yield a deep color causing characteristic long wavelength absorption band at 840 nm. The predominant chromagen with TCNQ is blue-colored radical anion, which probably resulted through the dissociation of an original donor-acceptor complex with the drug. This complex is formed by the lone pair of electron donated by the alendronate sodium as n-donor and the charge-transfer reagent as an electron acceptor, which a partial ionic bond (D^+^ A^−^) is assumed to be formed.

(1) $$\underset{\text{Donor}\;\text{Acceptor}}{\text{D}+\text{A}}\to \underset{\text{Donor-acceptor complex}}{\left[\text{D}\to \text{A}\right]}\to \begin{array}{c} \underset{\text{Radical anion}}{{\text{D}}^{+}+{\text{A}}^{-}} \end{array}$$

**Figure 1:**
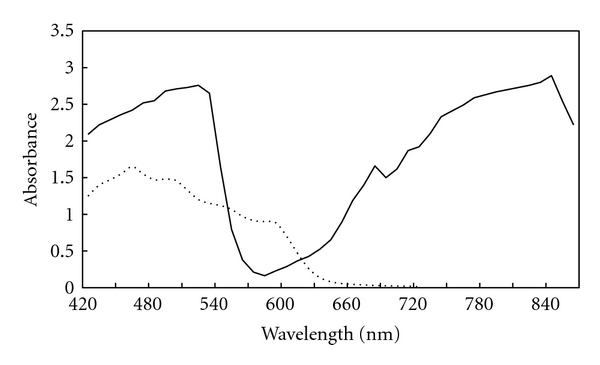
Absorbance spectra of the colored products produced from the reaction of alendronate sodium (5 *μ*g mL^−1^) with DDQ (*⋯*) and TCNQ (—).

The dissociation of the complex was promoted by the high ionizing power of acetonitrile solvent. DDQ is *π*-electron acceptor as a result of the strong electron withdrawing halo and cyano groups conjugated with the *π*-system [21, 22]. DDQ reacts instantaneously with basic nitrogenous compounds to form charge-transfer complexes of *n*-*π* type. The absorption spectrum of DDQ in methanol shows a characteristic band peaking at 360 nm. The addition of alendronate sodium solution to this solution causes an immediate change in the absorption spectrum, with a new characteristic band peaking at 465 nm. This band may be attributed to the formation of DDQ radical anions, which probably resulted from the dissociation of the donor-acceptor complex in a highly polar solvent like methanol. The maximum absorption of these charge-transfer complexes is shown in Figure 1. 

### 3.2. Stoichiometry and Mechanism of the Reaction

**Figure 2:**
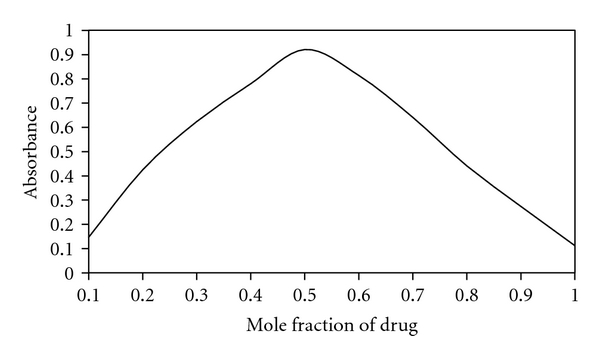
Continuous variation plot for the reaction of alendronate sodium and color producing regents.

**Scheme 1:**
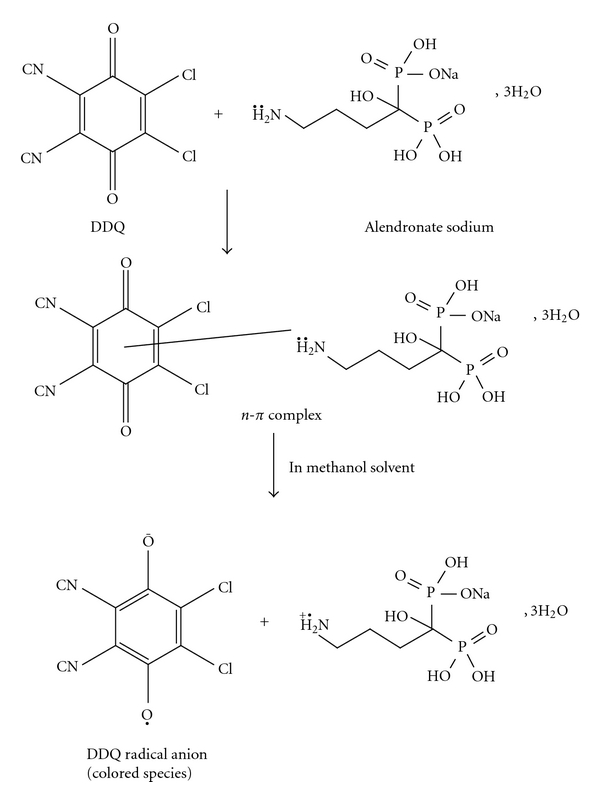
Reaction involved in analysis of alendronate sodium.

The stoichiometry of the reaction between alendronate sodium and each of TCNQ and DDQ was investigated by Job's method of continuous variation as described in experimental section under the head of *“Determination of the Molar Ratio”. *The mole ratio method suggested a donor to acceptor ration 1 : 1 (Figure 2) confirming the presence of one *n*-donating center in the alendronate sodium molecule (Scheme 1). 

### 3.3. Optimization of Reaction Conditions

The spectrophotometric properties of the colored species formed with TCNQ and DDQ as well as different parameters affecting the color development were extensively studied. The optimum conditions for the assay procedures (Methods A and B) have been established by studying the reaction as function of the concentration of the reagent, nature of the solvent, heating time, and stability of the colored products. 

#### 3.3.1. Effect of Color Producing Reagent and Time

For method A, the effect of volume of 1 mg mL^−1^ TCNQ solution was studied over the range of 2 10 *μ*g mL^−1^, in a solution containing 5.0 *μ*g mL^−1^ alendronate sodium. The results revealed the fact that 1 mL of TCNQ solution was required to achieve the maximum intensity of the color. Therefore 1 mL was the optimum value and maintained throughout the experiment. The reaction gets stabilized within the five minutes of heating at 70°C in water bath.

For method B, to study the influence of the volume of 5 mg mL^−1^ DDQ solution, we pipetted an aliquot of the drug solution containing 5.0 *μ*g mL^−1^ into a series of 10 mL volumetric flasks, followed by varying volumes of 1 mg mL^−1^ DDQ solution (2–12 *μ*g mL^−1^). The contents were diluted to the volume with methanol. The highest absorbance was obtained with volume 1.2 mL of 5 mg mL^−1^ DDQ solution. Further addition of DDQ solution cause-no change in the absorbance, so 1.2 mL was selected the optimum volume for all determinations. The intensity of the color formed on mixing the reagent reached maximum with in 40 seconds at room temperature and remained stable for two hours.

#### 3.3.2. Effect of Solvent

The polarity of the solvent used in the reaction between *π*-acceptors with *n*-donors can influence the formation of charge transfer complexes. Therefore, investigations were carried out to establish the most favorable solvent for the formation of the colored product. The solvents studied were acetone, dimethyl sulfoxide, N,N-dimethyl formamide, ethanol, methanol, acetonitrile, and isopropanol. Alendronate sodium was found to yield a colored product with TCNQ and DDQ when all these solvents were used.

However, acetonitrile was the choice solvent since that it gave maximum intensity and stability of color faster than the others in method A and methanol in method B.

### 3.4. Analytical Evaluation

TCNQ and DDQ were evaluated as a chromogenic reagent for spectrophotometric determination of alendronate sodium. Under the proposed experimental conditions, a linear response between absorbance and alendronate sodium concentration was verified. For method A, Beer's law was obeyed in a concentration range from 2 to 10 *μ*g mL^−1^ with correlation coefficient 0.998. The spectrophotometric method showed a molar absorptivity of 7.9 × 104 L mole^−1^ cm^−1^, indicating a good sensitivity for the samples analyzed.

While for method B, Beer's law was obeyed in a concentration range from 2 to 12 *μ*g mL^−1^ with correlation coefficient 0.9997 and molar absorptivity of 4.6 × 104 L mole^−1^ cm^−1^.

The regression equations for the described procedures were derived using the least-square method. The limit of detection (10.SD^blank^/slope of analytical curve) and limit of quantification (10.SD^blank^/slope of analytical curve) are shown in Table 1. The analytical parameters and the optical characteristics for the spectrophotometric determinations of alendronate sodium by the proposed methods are given in Table 1.

### 3.5. Interference Study

To study the potential interference problems from the commonly used excipients and other additives such as microcrystalline cellulose, anhydrous lactose, magnesium stearate, Croscarmellose, and calcium phosphate, recovery studies were carried out. Under the experimental conditions employed, to a known amount of drug (alendronate sodium 5.0 *μ*g mL^−1^), excipients in different concentrations were added and analyzed. Results of the recovery analysis are presented in Table 2. Excipients up to the concentrations shown in the Table 2 do not interfere with the assay. In addition, recoveries in most cases were around 100% and the lower values of the RSD indicate the good precision of the method.

## 4. Application

The applicability of the proposed methods for the determination of alendronate sodium in commercial dosage forms was examined by analyzing marketed products. The results of the proposed methods were statistically compared with reference method [23] and summarized Table 3. It is evident from the table that the calculated *t* and *F* values [24] are less than the theoretical ones at 95 % confidence level, indicating no significant difference between the methods compared. The proposed methods are sensitive, simple, accurate, and extraction free and are successfully applied for the quality control of pure alendronate sodium in pharmaceutical dosage forms.

## 5. Conclusion

The proposed spectrophotometric methods are simple, sensitive, rapid, and low-cost, do not involve any pretreatment or extraction steps and gives precise and accurate results. The proposed methods were successfully applied to analysis of alendronate sodium in tablets suggesting its use as a reliable and advantageous alternative to other previously reported methods for routine analysis of alendronate sodium in these samples.

## Figures and Tables

**Table 1:** Selected spectral data for the determination of alendronate sodium by proposed spectrophotometric methods.

Parameter	Values for method A	Values for method B
*λ*_max_ (nm)	840	465
Beer's law verification range (*μ*g mL^−1^)	2–10	2–12
Molar absorptivity (L mole^−1^ cm^−1^)	7.9 × 104	4.6 × 104
Sandell's sensitivity (*μ*g ml^−1^ per 0.001 A)	4.0 × 10^−3^	4.9 × 10^−3^
Regression equation (Y*)		
Slope (b)	0.0254	0.0351
Intercept (a)	0.0238	0.0241
Correlation coefficient (*r*)	0.9989	0.9997
Limit of detection (*μ*g mL^−1^)	0.19	0.30
Limit of quantification (*μ*g mL^−1^)	0.62	0.99

Y*: a + bC, where C is the concentration of analyte (*μ*g/mL) and Y is absorbance unit.

**Table 2:** Determination of alendronate sodium in the presence of possible excipients (5 *μ*g mL^−1^ of alendronate sodium was taken for interferences studies).

Excipient	Amount taken (g mL^−1^)	% Recovery ± RSD (*N* = 5) method A	% Recovery ± RSD (*N* = 5) method B
Microcrystalline cellulose	300	99.6 + 0.25	99.6 + 0.25
Anhydrous lactose	300	99.5 + 0.25	99.5 + 0.25
Magnesium stearate	200	100.1 + 0.30	100.1 + 0.30
Croscarmellose sodium	100	99.8 + 0.40	99.8 + 0.40
Calcium phosphate	50	99.4 + 0.45	99.4 + 0.45

**Table 3:** Determination of alendronate sodium in pharmaceutical formulations by the proposed and reference [23] methods.

Sample	Recovery ± S.D.		
	Official method	Method A	Method B
Fosamax tablets	99.60 ± 0.83	99.75 ± 0.78	99.87 ± 0.69
*t*		0.24	0.60
*F*		1.15	1.14
Ostepor tablets	100.02 ± 0.46	99.56 ± 0.53	99.92 ± 0.53
*t*		1.24	0.54
*F*		1.58	1.28
Bongard tablets	99.75 ± 0.63	99.90 ± 0.41	100.05 ± 0.61
*t*		1.42	0.52
*F*		0.75	1.13
Bonate tablets	100.12 ± 0.52	100.01 ± 0.57	99.89 ± 0.68
*t*		1.28	0.34
*F*		1.22	1.12

*Average of 3 independent analyses.
